# Rethinking Elective Cataract Surgery Diagnostics, Assessments, and Tools after the COVID-19 Pandemic Experience and Beyond: Insights from the EUROCOVCAT Group

**DOI:** 10.3390/diagnostics10121035

**Published:** 2020-12-02

**Authors:** Daniele Tognetto, Antoine P. Brézin, Arthur B. Cummings, Boris E. Malyugin, Ozlem Evren Kemer, Isabel Prieto, Robert Rejdak, Miguel A. Teus, Riikka Törnblom, Mario D. Toro, Alex L. Vinciguerra, Rosa Giglio, Chiara De Giacinto

**Affiliations:** 1 Eye Clinic, Department of Medicine, Surgery and Health Sciences, University of Trieste, 34134 Trieste, Italy; alexluciavinciguerra@libero.it (A.L.V.); giglio.rosam@gmail.com (R.G.); chiaradegiacinto@gmail.com (C.D.G.); 2 Université de Paris, Hôpital Cochin, 75014 Paris, France; antoine.brezin@aphp.fr; 3 Wellington Eye Clinic, D18 T8P3 Dublin, Ireland; abc@wellingtoneyeclinic.com; 4 S. Fyodorov Eye Microsurgery Federal State Institution, Russian Federation, 127486 Moscow, Russia; boris.malyugin@gmail.com; 5 University of Health Sciences, Ankara City Hospital, 06800 Ankara, Turkey; ozlemvidya@gmail.com; 6 Department of Ophthalmology, Fernando Fonseca Hospital, 2720-276 Amadora, Portugal; isabelmcprieto@gmail.com; 7 Department of General Ophthalmology, Medical University of Lublin, 20-079 Lublin, Poland; robertrejdak@yahoo.com; 8 Department of Ophthalmology, University of Alcalá, 28802 Madrid, Spain; miguelteus@gmail.com; 9 Department of Ophthalmology, TYKS Hospital, 20521 Turku, Finland; riikka-maija.tornblom@tyks.fi; 10 Faculty of Medical Sciences, Collegium Medicum, Cardinal Stefan Wyszyński University, 01-815 Warsaw, Poland; 11 Department of Ophthalmology, University Hospital of Zürich, University of Zürich, 8091 Zürich, Switzerland

**Keywords:** cataract surgery, phacoemulsification, COVID-19 outbreak

## Abstract

The progressive deterioration of the visual function in patients on waiting lists for cataract surgery has a negative impact on their quality of life, especially in the elderly population. Patient waiting times for cataract surgeries in many healthcare settings have increased recently due to the prolonged stop or slowdown of elective cataract surgery as a result of coronavirus disease 19 (COVID-19). The aim of this review is to highlight the impact of such a “de-prioritization” of cataract surgery and to summarize some critical issues and useful hints on how to reorganize cataract pathways, with a special focus on perioperative diagnostic tools during the recovery phase and beyond. The experiences of a group of surgeons originating from nine different countries, named the European COVID-19 Cataract Group (EUROCOVCAT), have been combined with the literature and recommendations from scientific ophthalmic societies and healthcare institutions. Key considerations for elective cataract surgery should include the reduction of the number of unnecessary visits and examinations, adoption of precautionary measures, and implementation of telemedicine instruments. New strategies should be adopted to provide an adequate level of assistance and to guarantee safety conditions. Flexibility will be the watchword and regular updates would be necessary following scientific insights and the development of the pandemic.

## 1. Introduction

The most prevalent ophthalmic diseases in economically developed countries are those of the ageing eye (glaucoma, age-related macular degeneration, and cataract). In Europe, the prevalence of cataract is 64% for the population over 70 years and it increases with age, with higher rates in Germany and Italy [1]. In many countries, cataract surgery is one of the most commonly performed procedures and it has proven to be one of the most cost-effective health-care interventions [2,3]. Indeed, cataract related visual impairment has effects not only on daily life activities, but also on one’s psychological wellbeing.

Cataract surgery has been shown to improve the cognitive input in Alzheimer disease and in other forms of dementia referred to as “depressive pseudodementia”, a functional disturbance associated with a depressive mental status [2,3,4]. Ishii et al. reported a significant improvement in vision-related quality of life, cognitive function, and depressive mental status in elderly patients before and after bilateral cataract surgery [5]. Moreover, a National Bureau of Economic Research study found cataract surgery to be one of the main treatments, together with cardiac care, contributing to an increase of 1.8 years in healthy life expectancy at the age of 65 years [2]. Tseng et al. analyzed the risk of hip fracture within 1 year in a cohort of patients diagnosed with cataract, aged 65 years and older. The authors reported lower odds in those who had cataract surgery compared to those who had not undergone the surgery [6].

In last few decades, the demand for cataract surgery has progressively increased because of different factors such as population ageing, earlier intervention referral, and a higher frequency of second eye surgery [7]. Unfortunately, waiting times and limited access to cataract surgery represent an important burden on countries that are primarily based on public funded healthcare systems [3]. Recently, the situation has been worsened by the prolonged cessation of elective procedures due to the outbreak of coronavirus disease 2019 (COVID-19) [8,9,10,11,12].

In some European settings, a 97% reduction in cataract surgery volume has been reported between March and April 2020 compared to the same period in 2019 [9].

Forecasting the spread of COVID-19 or the end of the pandemic represents a challenge [13]. Therefore, it is important to plan robust solutions to meet the expected increased demand for cataract surgery, even in the case of a persistence of infection risk during the next months or years [7,14,15]. Most patients attending the Ophthalmic Outpatient Departments (OPDs) are aged 65 years and older and are considered at a higher risk for worse outcome in case of Severe Acute Respiratory Syndrome Coronavirus 2 (SARS-CoV-2) infection [16,17]. Thus, for cataract surgery pathways, it should be mandatory to rethink patient management, including preoperative and postoperative care diagnostic tools in order to maintain safety conditions for patients and healthcare staff.

Here we summarize some practical hints and key steps for the reorganization of cataract care and surgery during the time of COVID-19, merging the experiences of the European COVID-19 Cataract Group (EUROCOVCAT) and the most updated literature and guidelines.

## 2. Materials and Methods

An extensive search of peer-reviewed literature using the PubMed database was performed. The search date was limited to 31 December 2019–17 November 2020. For the literature search, each of the following terms was used, always in combinations with “SARS-CoV-2” or “COVID-19”: “ophthalmology”, “cataract”, “cataract surgery”, “cataract extraction”, “recommendations”, “telemedicine”. Two reviewers (R.G. and A.L.V.) independently evaluated the list of articles in terms of titles and abstracts (if available) to identify relevant articles to the topic (key points and burdens for resuming elective cataract surgery during the time of the COVID-19 pandemic). The full texts and associated reference lists of relevant articles underwent further evaluation for eligibility by the same reviewers. Any disagreement was assessed by consensus and a third reviewer (M.D.T.) was consulted when necessary. Only English language articles were selected. Review articles, original research, and letters presenting original data were included. Articles reporting experiences from other specialties or dealing with children and young patients were excluded.

For recommendations and guidelines analysis, publicly-available data displayed on the official websites of some national and international scientific ophthalmologic societies and international healthcare institutions were extracted, translated into English when applicable, and summarized by a working group comprised of expert ophthalmic surgeons coming from nine different European countries [9,18]. The group (EUROCOVCAT) was born with the purpose of sharing skills, experience, perspectives, and proposals that emerged during the troubling time of the COVID-19 pandemic. Input was also provided by representatives from other areas (laboratory medicine and healthcare management) who were invited to review the work. Since March 2020, monthly discussions have been organized through conference calls to exchange and debate ideas related to practice reopening and available guidelines. It is important to note that guidelines are subject to change and this paper reflects the respective status as of the date they were discussed during the group conference calls (date of access reported in the reference list).

## 3. Results

The search identified a total of 887 unique articles. After the two reviewers completed screening, 714 were excluded because the contents in the title and/or abstract were not relevant for this review. The remaining 173 full text articles were assessed for eligibility. Subsequently, 91 articles were removed after a full-text review. Finally, 82 unique articles were included in qualitative analysis. For the guidelines and recommendations analysis, data from the following societies and institutions were included: The American Academy of Ophthalmology (AAO), Royal College of Ophthalmologists (RCOphth), Società Oftalmologica Italiana (SOI), Sociedad Española de Oftalmología (SEO), Société Française d’Ophthalmologie (SFO), World Health Organization (WHO), and European Centre for Disease Prevention and Control (ECDC).

### 3.1. Ophthalmology Practice and Infection Risk

Many reports have underlined the specific risks of SARS-CoV-2 infection in ophthalmology [19,20,21,22,23,24,25,26,27,28]. The ophthalmic examination requires close, face-to face contact with patients, and the risk of viral shedding from the respiratory tract might represent a substantial issue for eye care practitioners [19,20,25,26,28,29,30,31]. Although droplets appear to be mainly responsible for viral transmission, the infectious risk of ocular tissues and fluids has been debated [32,33,34,35,36,37,38,39]. SARS-CoV-2 RNA has been detected in ocular fluids, even without any signs of conjunctivitis [34,35,36]. Seah et al. have analyzed 64 tear samples of 17 COVID-19 patients (without any sign of conjunctivitis at presentation) and found no positive samples for SARS-CoV-2 [33]. In a case series by Xia et al., in 60 conjunctival swabs from 30 patients with SARS-CoV-2, only two (coming from the same patients) were positive for viral RNA [38].

In a recent work by Shemer et al., all conjunctival swab tests performed respectively on 16 patients with confirmed cases of COVID-19 (with and without ocular involvement) and on 32 controls, showed negative results for three viral genes tested (E, N, and RdRp) [40]. A recent meta-analysis reported pooled sensitivity of ocular tissue/fluid in detecting SARS-CoV-2 to be only 0.6% in comparison with nasopharyngeal and sputum swabs. The relatively low rate of ocular samples tested positive could be consistent with a limited viral load in ocular tissues/fluids [41]. Furthermore, the viability of viruses might be influenced by the collection technique, the type of swabs—the presence of calcium alginate could inactivate the virus and inhibit polymerase chain reaction (PCR) tests—and the use of topical anesthesia for sample collection [42].

Clinical reports have suggested a variable incidence of conjunctivitis in patients showing SARS-CoV-2 positivity, from 0.9% up to 31.6% [39,43,44]. As recently underlined by Napoli et al., current available articles have not reported the use of concomitant topical medications that might explain conflicting data regarding ocular tissues involvement [45]. The authors have observed that many topical ophthalmic medications (artificial tears, anti-glaucoma medications, miotics, anti-inflammatories, and antimicrobials) could have, as a secondary effect, an antiviral action. The antiviral side effect (not an adverse effect) could be explained by the ingredients or excipients contained in the drops or ointment such as benzalkonium chloride (BAK), sodium perborate chlorobutanol, citric acid, disodium-ethylene diamine tetra-acetate (EDTA), and boric acid. They analyzed different classes of drugs with broad antiviral activity (including against coronaviruses) suggesting a repurposing potential for many of them [45]. However, no specific topical antiviral is currently available for SARS-CoV-2.

The broad-spectrum antimicrobial effect of povidone-iodine (PVI) is well known [46]. In ophthalmology, it is commonly used for infection prophylaxis prior to intraocular surgical procedures. Many reports have described PVI antiviral efficacy against various types of viruses, adenoviruses and coronaviruses included [46,47,48,49]. The virucidal activity of PVI against Middle East respiratory Syndrome Coronavirus (MERS-CoV) and SARS-CoV has been tested in vitro with different concentrations and time of exposure [50,51]. Kariwa et al. reported PVI products (concentration ranging from 0.23% up to 1%) to reduce the SARS-CoV viral counts to undetectable levels within 2 min of exposure [51]. In a work by Contini et al., the use of PVI eyedrops (concentration and treatment regimen not specified) has been proposed as auto-prophylaxis during the early phase of the disease in COVID-19 patients with conjunctival congestion [52]. Edington et al. have suggested that the use of a routine perioperatively PVI ought to be recommended in ophthalmology protocols for safe ocular surgery during the COVID-19 pandemic, although its use should not substitute the requirements for other safety measures, PPE (personal protective equipment) included [53]. Madan et al. suggested the use of 1 mL of 5% PVI (Betadine) diluted with 4 mL of lubricant drops containing BAK to reduce patient discomfort (burning and irritation) while enhancing the antiviral action [47]. A “multifocal” PVI application (oral gargles, 1% PVI conjunctival application for 3 min and 0.4% PVI lacrimal system irrigation) has been proposed in lacrimal system surgeries to reduce the viral load in aerosols that are inevitably generated [54]. Obviously, the lacrimal system surgery is a high-risk procedure because it requires the contact not only with the ocular surface and tears but also with the nasal mucosae. For cataract surgery, the current conjunctival application of 1–5% PVI for 3 min might provide an adequate reduction of viral load, but further in vitro and in vivo studies would be necessary to validate the anti-viral effect against SARS-CoV-2 and the most adequate concentration and administration regimen [47,49].

### 3.2. Medical Liability during COVID-19 Pandemic

When considering the risk/benefit balance of whether to resume elective activities, health and legal protection is paramount for healthcare providers and patients [55]. Several issues exist including (but not limited to) infected health practitioners who were not given adequate PPE, patients claiming SARS-CoV-2 infection transmitted in a hospital setting, and healthcare institutions and their management made responsible for the worsening of non-urgent diseases [56,57]. It should be remembered that liability provision should safeguard healthcare workers (HCWs) and it should not be used by health institutions as an escape to avoid responsibilities [56]. It appears that ordinary law might be inadequate in the unique context of a pandemic and that the standard of care (SOC) should be adapted for pandemic conditions [58]. Medical liability is strictly related to the concept of SOC and the failure to meet such a standard (negligence) [59]. However, SOC might significantly vary in many countries or even within cities, depending on the levels of resources available. In some federal states of the United States, exceptional or temporary laws have been introduced assuring providers’ immunity from civil and/or criminal liability, with the exception of obvious circumstances (gross negligence, willful misconduct, etc.) [60,61]. In different European countries, new laws or amendments have been proposed for medical liability protection, but ordinary law is generally still applied [56]. Although in many countries the suspension of elective procedures and the treatment delay of conditions other than COVID-19 were in response to government orders to restrict non-emergency procedures, during the following months and years a rise in legal claims might occur. The medico-legal implications might be different and complex and go beyond the aim of the present work.

Governments in Europe are in various stages of re-opening and at the time we are writing (17 November 2020) many countries are facing a new increase in COVID-19 cases. Despite scientific efforts and different infection control measures put in place, ups and downs in the number of cases might be expected in the near future [62]. Thus, we cannot plan to resume elective activities without implementing a safe pathway [63]. Healthcare facilities and institutions should be responsible for the adoption, at an organizational and structural level, of preventive measures to avoid the risk of cross-infection inside hospitals. Interim recommendations from scientific societies and institutions are useful but they are evolving rapidly. National and local public health institution websites should be checked regularly for updates and to implement practices, as necessary. Local risk management might require an expansion of insurance coverage and insertion of a reference to COVID-19 risks in informed consent forms [56,64].

In any case, providers should document and periodically review their protocols and other actions taken to comply with relevant guidance. If compliance to applicable guidelines cannot be provided, elective care resumption should be deferred because no immunity provision is likely to provide protection in that circumstance. Unfortunately, general measures (use of PPE, limited access for visitors, social distancing, HCWs testing for SARS-CoV-2,) although mandatory are not sufficient to set to zero the risk of infection in the wards. Many challenges remain unsolved and they will require careful development and implementation [56,61].

### 3.3. Appropriateness and Prioritization Tools for Cataract Surgery

COVID-19-related backlog on cataract waiting lists has once again placed attention on an unsolved problem: The need for measurement instruments for assessing appropriateness and prioritization for cataract surgery. In the context of the current pandemic, the need to integrate the assessment of patient readiness and aspects of quality of life into clinical practice has become even more pronounced than before [57,65]. If fully implemented, this could mean an important step forward for value-based healthcare [66,67]. Different tools for the prioritization of cataract patients have been proposed in several countries (i.e., Canada, UK, Sweden, Spain, and New Zealand) [68,69,70,71,72,73,74,75]. Some of them combine clinical data (visual acuity and symptoms) with the social impact of the disease (self-care, driving) [67]. The Western Canada Waiting List Project (WCWLP) is one of the most successfully validated systems and has been used as the basis for developing other systems. It is currently used by Alberta Health Services in Canada. Many other examples of measurement instruments have been reported, although reliability for some of them have not yet been tested. The RCOphth has proposed a tool combining ocular and systemic clinical data with a subjective measure of visual disability assessed through six simple questions [67].

Starting from the chronological order, patients’ willingness for surgery should be re-checked [76]. When considering age, different aspects should be evaluated. Although priority might be given to younger patients because of working needs, a further delay of surgery might have a negative impact for the elderly in terms of life expectancy. Pre-existing medical conditions (such as diabetes, high blood pressure, heart disease, cerebrovascular disease, lung disease, or cancer), have been associated with worse outcomes in the case of SARS-CoV-2 [8]. Thus, the presence of comorbidities, especially in elderly patients, should be considered [66,67]. Ocular comorbidities or special conditions such as monocular patients (both anatomical or functional), active proliferative diabetic retinopathy, or any macular lesion that requires additional examinations with clear lens, anisometropia, or high refractive errors might be listed among items contributing to the priority final score [68,70,71,73]. Second eye surgery could be attributed as a lower priority on lists [77]. Further studies might be necessary to validate and establish the appropriateness, reliability, and suitability of available instruments and criteria in different settings.

### 3.4. SARS-CoV-2 Screening

The three main types of detection assays for SARS-CoV-2 include nucleic acid tests (NAATs, which detect the presence of viral RNA), antigen tests (that detect the presence of a viral antigens), and antibody tests (that detect the presence of antibodies generated against SARS-CoV-2). Serology tests are suggested to identify individuals who have overcome the infection, developing an immune response rather than to detect new cases [78,79,80]. Both NAATs and antigen tests can be used to detect ongoing infection and WHO’s interim guidelines specify using a NAAT to confirm COVID-19 cases [79]. Samples for diagnostic tests for SARS-CoV-2 can be taken from the upper (nasopharyngeal/oropharyngeal swabs, nasal aspirate/wash, or saliva) or lower respiratory tract (sputum, tracheal aspirate, or bronchoalveolar lavage). Even though many reports have described the presence of viral particles in tears and in ocular tissues, conjunctival swabs might have a low chance of detecting the virus because, as previously reported for SARS-CoV, viral secretion might be seen only during the early phase of the disease and because only a small number of exfoliated cells are generally collected by the swab [5,13]. Moreover, the viability of the virus and/or the PCR tests might be influenced by different factors such as the use of topical anesthesia for sample collection (see Section 3.1. “Ophthalmology practice and infection risk”) [23].

Nucleic acid tests typically use an amplification step based on reverse transcriptase polymerase chain reaction (RT-PCR). The sensitivity may vary depending on the type of specimen [79]. In confirmed cases of people with COVID-19, nasopharyngeal swab tests have shown a higher sensitivity compared to saliva testing (98% vs. 91% respectively) [81]. The combination of nasopharyngeal/oropharyngeal swab samples has proven to be more sensitive for the diagnosis of SARS-CoV-2 compared to only the nasopharyngeal swab [42,82]. Saliva could be considered as an alternative specimen that can also be employed for self-sampling in case the above-mentioned specimens are not easily collected [81]. However, these data have been derived from studies performed on symptomatic patients. Moreover, another limitation of the use of RT-PCR as a screening tool for asymptomatic patients is that viral replication time might take up to 14 days after exposure to achieve the threshold for viral RNA to be detected [83].

In addition to testing, alternative strategies that include self-isolation and other distancing measures might be needed to enhance outcomes. Preoperative screening programs should aim to improve the safety of patients and HCWs, outcomes, and resource management (i.e., PPE) [83,84,85]. HCWs should be periodically tested to reduce the risk of nosocomial transmission to fragile patients, other staff members, and from the hospital to the community [72]. The most recent ECDC recommendations indicate to test patients before planned hospital admission, including elective surgery 24–72 h before admission [77]. However, for cataract surgery, admission is generally not required and the total amount of time spent at hospital is quite limited. The necessity of preoperative laboratory screening could be carefully evaluated according to local risk [67,77,86,87].

Since cataract is generally performed under topical or local anesthesia and is commonly integrated in a fast-track approach, some centers might opt to not require screening laboratory tests for infection, deciding to manage patients as potential SARS-CoV-2 carriers according to the local risk of infection and resources. Where and when it is feasible or required by local provision, the pre-operative SARS-CoV-2 screening should be integrated into the one-stop pre-surgical assessment [67,77]. Whether or not any testing is performed, a careful triage (the day before surgery and/or at check-in the day of surgery, see below) is highly recommended [88]. As for the pre-operative assessment in the presence of infection risk, it is advisable to postpone the surgery [67,77].

### 3.5. Preoperative Assessment Reorganization

Although COVID-19 transmission occurs mainly via droplets, the presence of viable SARS-CoV-2 particles in aerosols for multiple hours has been reported [19,25]. Thus, it is suggested that appointment schedules are reviewed. The interval between each ophthalmic procedure (including surgeries) should be lengthened due to additional time required for triage and infection risk assessment, adequate room aeration (ventilation rate time), and sanitization procedures [89,90,91]. For this reason, it is advisable to extend the medical activities at a wider range of hours reviewing the work shifts of medical, nursing, and administrative staff. All outpatient services should be equipped with protective slit lamp breath shields, disposable devices including tonometer tips, and single-dose eye drops [27,67,92]. Pneumotonometry and air-puff tonometry should be avoided because of the risk of the aerosolization of air particles [93]. Furthermore, diagnostic hubs should be created in cooperation with primary and secondary care providers, in a regional or local network, to manage the referral and post-operative care of patients [67].

#### 3.5.1. Patient’s Arrival

At their arrival, all patients attending facilities should undergo a series of precautionary procedures including: Disinfection of their hands with hydroalcoholic gel, wearing surgical mask provision, body temperature measurement, administration of a questionnaire on the current state of health, and completion of a self-declaration regarding the awareness of the risk of contagion [40,54]. It is suggested to perform these check-in procedures in a dedicated “filter” area, placed outside the ward. In the presence of infection risk (alarming symptoms and/or body temperature ≥ 37.5 °C), detected during telephone pre-triage or at the time of patient’s arrival, the appointment should be rescheduled and the patient should be instructed to self-isolate and to contact their general practitioner or the toll-free number provided by local authorities. If the patient requires an accompanying person, all these instructions should be given to the caregiver as well [67,93,94,95].

#### 3.5.2. Pre-Surgical Assessment

The main goal for preoperative assessment should be to limit the number of visits and amount of time spent by patients at a facility. Whenever possible, one-stop preoperative assessment and surgery should be preferred for uncomplicated cases [67,94]. The workflow system described by Gabbay et al. has shown that most cataract patients could be scheduled for surgery based on referral letters, with surgery done immediately following preoperative examination [96]. In this system, a senior ophthalmologist reviews the referral letter of the community-based ophthalmologists who are instructed to report past medical and ocular history, visual acuity, intraocular pressure, anterior segment examination, and dilated fundoscopic examination. With this preoperative ophthalmologic triage system, a preoperative evaluation at the ophthalmology clinic or referral center is reserved only for those with incomplete referral letters, with discrepancies between visual acuity and described findings, or for patients with a history (systemic or ophthalmologic) suggesting an expected higher surgical risk [96].

In order to prevent day-of-surgery cancelation of cases and to limit the number of patients, it has been suggested to perform a preliminary evaluation to assess visual expectations and special needs such as presbyopia correcting intraocular lenses [67,94]. This evaluation could be performed by phone or providing interactive forms to be completed on patient devices (tablets, PCs). In any case, a telephone triage is recommended the working day prior to the pre-surgical assessment and/or surgery to identify whether patients are potentially SARS-CoV-2 infected. The telephone triage might be important to avoid the admission of positive or suspected patients into working areas [67,94]. The triage should include targeted questions. In case of immunosuppression, it is advisable to organize the examination so as to isolate the patient and ensure maximum protection [67,94]. At the time of the telephone triage, patients should be invited to come alone to the visit unless strictly necessary (minors, disabled patients, or linguistic barrier), and to respect the visiting time assigned, to avoid crowding of people. Moreover, patients should be informed of unavoidable risk of accidental SARS-CoV-2 infection when coming to OPDs, despite all precautionary measures adopted to prevent this risk [67,94].

Preoperative routine medical testing (require blood tests, chest X-rays, and electrocardiogram preoperatively) has not been shown to influence the surgical outcome or rate of adverse events (cardiac attack or death) and thus, unless for selected cases, could be avoided [97].

During the pre-operative visit, it would be advisable to limit the number of instrumental examinations to those strictly required for surgery (biometry, topography, and endothelial specular microscopy) [67,94].

It has been reported that up to 17% of cataract patients might have some macular findings not detected during fundus examination. The cost-effectiveness of routine baseline optical coherence tomography (OCT) is debated [97,98,99,100,101]. OCT prior to cataract surgery might be useful in patients with a visual decrease not explained by the degree of cataract or in patients with a positive history of retinal pathology or candidates to premium IOLs implantation [97,98,99,100,101]. The optical biometer (IOL Master 700—Carl Zeiss Meditec AG, Jena, Germany) based on swept-source optical coherence tomography (SS-OCT) technology performs a small central macular scan, introduced as the quality control of the patient’s fixation during the examination. It has been reported that this macular scan has a positive and negative predictive value of 0.78 and 0.86, respectively. Thus, next generation optical biometers based on SS-OCT could be a useful screening tool although conventional SD-OCT remains mandatory to rule out the presumed diagnosis [14].

In any case, all preliminary procedures should be completed in one visit during pre-surgical assessment. Informed consent should be updated as the risk connected to SARS-CoV-2 and patients should be instructed accordingly [67,102,103].

### 3.6. Key Points for the Surgery

Pupil dilation might be done by the patient, adequately instructed at the time of pre-surgical assessment, or by intracameral injection in the operating room (OR) during surgery. The use of a manufactured intracameral combination of 2 mydriatics and 1 anesthetic for mydriasis and anesthesia might be an option to limit contact with the conjunctiva and tear film during patient preparation [104,105,106]. Communication with the patient during the surgery should be minimized. The number of people attending the OR should be kept to the necessary minimum (i.e., one surgeon and two nurses) [67,77]. It might be reviewed when concerning the need for other staff members including the anesthetist, assistants, and residents in training. Bilateral same day sequential surgery might be an option to be considered in alignment with local legislations and surgeon preferences. An immediately sequential bilateral cataract surgery could be advised in patients at low risk of intraoperative and/or post-operative ocular complications, in patients at increased risk of systemic complications or distress but requiring general anesthesia for cataract surgery, and if surgery in the fellow eye is expected early [67,77,107]. Additionally, a combined procedure with trabeculectomy or vitrectomy should be preferred in all cases of co-existing pathologies that require further surgical treatment [77].

It has been debated whether the rapid oscillations of the phacoemulsification probe could generate aerosols. In a laboratory study on goat and cadaveric human eyes Shetty et al. found no visible aerosol generation during standard phacoemulsification [108]. McGhee et al. recorded microdroplets and spatter contamination on the surgeons’ gloves and gown during the phacoemulsification of porcine eyes [109]. Darcy et al. reported differences in aerosol generation according to the phaco tip size (2.2 mm vs. 2.75 mm) with no aerosol being generated with a 2.2 mm tip [110]. Although there might be some aerosol being generated during phacoemulsification, considering that the potential viral load of the ocular surface might be virtually eliminated using a topical application of diluted povidone iodine and that the aqueous humor should be completely replaced by sterile balanced saline solution (BSS) during phacoemulsification, the risk for infection spread seems to be very low during cataract surgery [110,111]. Nevertheless, the surgeon might consider using hydroxypropyl methylcellulose 2% (HPMC) to keep the cornea lubricated and incision sealed, decreasing the amount of aerosol generation during phacoemulsification [110].

A general consensus on the use of PPE during elective ophthalmic procedures is still lacking [27,88,102,112]. The OR staff should wear surgical masks (unless suspected cases), gloves, and should be trained about wearing and taking off PPEs. The surgeon and scrub nurse might be more exposed and for a longer time to potentially infective viral sources from biological fluids (droplets and or aerosol generated during the surgery) [76]. The AAO and RCOphth have recommended protection for the mouth and nose (e.g., filtering face piece respirator masks) and eyes (e.g., goggles, face-shields) [67,112]. The use of higher order PPE (i.e., FFP2/3) might be an option, in particular when pre-surgical SARS-CoV-2 screening is not available, however the choice of mask type would generally follow internal institutional protocols [27,90,112,113]. Some of these measures such as goggles, although mandatory, might impede focusing the microscope. A three-dimensional (3-D) heads-up display system could be of help in avoiding the need to look through the surgical microscope [55,114].

Single use equipment should be preferred and instruments should be covered properly. Special semi-transparent ophthalmic drapes or protecting shields for operating microscopes might be adopted to reduce the risk of viral transmission, although these measures do not replace the use of adequate PPE [66,80].

### 3.7. Postoperative Assessment

The number of post-operative assessments should be kept to a minimum and patients should not be asked to come to the hospital unless in case of complications or high-risk ocular comorbidities [115]. For cataract surgery, different practice patterns exist worldwide regarding post-operative care [116]. According to the AAO and RCOphth, an in-person first-day postoperative visit should not be routinely performed except in functionally monocular patients, in case of intraoperative complications, or in patients at a high risk of immediate postoperative complications (e.g., intraocular pressure spikes) and/or with ocular comorbidities [117,118]. Timing and even the need for an ophthalmic follow up after cataract surgery have been debated.

In a systematic review and meta-analysis of three randomized clinical trials (886 patients), Kessel et al. reported no increased safety obtained by reviewing patients on the first postoperative day in comparison with 2 weeks postoperatively, with no differences in final visual acuity and in the number of unscheduled visits. The authors recommended a deferment of the early postoperative review in low-risk patients (non-glaucomatous patients with no intraoperative complications and operated by experienced surgeons) [119]. A prospective non-randomized cohort study (256 eyes of 238 patients) by Tal and co-workers showed it was safe to substitute the first day post-operative visit with a telephone survey after routine phacoemulsification [120]. Allan et al. analyzed the clinical intervention rate occurring during 1652 routine follow-up visits after uncomplicated phacoemulsification. They found an intervention rate of 2.8%. The intervention rate was higher (50%) for unscheduled emergency service visits (7.3% of patients). They suggested as an alternative to routine postoperative follow-up examinations, shared care with non-ophthalmologists and improved perioperative patient education for self-referral [121].

Eloranta et al. retrospectively reviewed the outcomes of 1628 patients to evaluate the potential benefit of a routine 1-month post-operative visit after cataract surgery. They analyzed two cohorts of patients who underwent cataract surgery in 2006, when at least a 1-month follow-up visit was advised for all patients and in 2009 when patients, with the exception of selected cases, were not scheduled for a routine post-operative check-up. In both cases, patients were instructed to contact the department if they experienced pain, vision deterioration, or ocular discharge. Data including perioperative complications, ocular comorbidities, scheduled and unscheduled visits, and the need for a surgical or medical intervention were collected regarding the first 5 years of follow up. They found no issues attributable to the lack of a 1-month check-up during the follow up, with only 0.5% of patients in the 2006 cohort, and 0.3% of patients in the 2009 cohort requiring hospital referral. The authors concluded that a postoperative check-up visit might not be required in most cases with the exception of the presence of comorbidities and/or complicated cases [122]. For routine cases, an option could be a 1-day post-operative telephone consultation followed by a 4-week post-operative visit performed in cooperation with a patient’s primary eye care and community ophthalmology services [67,103,123,124,125]. Nowadays, teleophthalmology represents not only a research tool but has evolved into a clinical service [126,127]. The gap in healthcare coverage caused by the COVID-19 pandemic has highlighted the need for its rapid implementation. Although different digital approaches have proven useful for cataract patient screening, only fewer reports have evaluated telemedicine for eye examination following cataract surgery [128,129,130,131,132,133]. In a case series by Smith et al., post-operative video-examination showed reliability in detecting oedema at the central cornea but they did not consistently detect anterior chamber flare and failed to detect Descemet membrane folds or anterior chamber cells [128]. Despite a great number of applications being commercially available for visual function testing, some limitations have been reported including costs, restricted availabilities only on certain devices, set-up requirements, and modest agreement between application-based measurements and office-measured visual acuity [134,135,136,137]. Williams et al. from the Ophthalmology Department of the University of Pittsburgh Medical Center developed a video visit workflow that has proven useful in the management of a range of ophthalmic complaints. The authors designed and deployed an online eye chart available online that is suitable not only for visual acuity testing but also for Amsler grid and desaturation testing [25,138].

Teleconsultation-phone calls and video assessments, among different telemedicine tools, might be an option whenever available but major gaps still exist (i.e., the learning curve in the older population, intraocular pressure, and anterior chamber flare evaluation). It is advisable that cooperation should exist between governments and vision-related organizations so as to deploy new networks and digital resources [133]. In any case, independent of the options selected for follow up, patients should be informed and instructed to contact their ophthalmology unit in case of warning symptoms or signs and a dedicated contact route should be available and accessible accordingly [67,103].

## 4. Discussion

The COVID-19 global pandemic has caused many changes in healthcare worldwide creating a backlog and raising new challenges for physicians, ophthalmologists included. Vision loss is one of the most feared disabilities and it represents an important public health issue. Vision loss has a significant impact on individuals and society in terms of morbidity, quality of life, and costs [139]. Early diagnosis and prompt management of the most common and treatable eye conditions, including cataracts can prevent or slow down vision loss [139,140,141,142,143]. The significant physical and psychological benefits of cataract surgery should be a powerful reminder of the value of ophthalmic interventions, especially for older adults and those suffering from other health problems [4]. With this manuscript, the EUROCOVCAT group would like to underline the importance of the need to rethink cataract surgery pathway to guarantee sufficient access to cataract care in future. When dealing with waiting lists, the impact on quality of life should be one of the main factors to be considered. The mainstays for cataract surgery reorganization should include strict precautionary measures for those attending facilities, social distancing, adequate use of PPE, careful preoperative screening, reduced number of people in the OR, limited number of assessments, both preoperative and postoperative, and the implementation of telemedicine tools [10,19,67,94,103].

Many reports have underlined the need for screening patients for COVID-19 before a surgical or diagnostic procedure so as to reduce risk of cross infection and to prevent excessive use of limited supplies [84,85,144]. A symptom questionnaire to exclude COVID-19 would probably be the most important tool, with further screening through RT-PCR testing in selected cases and according to local epidemiological figures. Patients scheduled for cataract surgery represent a higher-risk group for infection, complications, and death from SARS-CoV-2 [8]. The use of pre-operative screening questionnaires and peri-operative testing of such a high risk group might provide dual benefits of protecting patients and HCW whilst supporting infection control [145]. New strategic public health approaches are advisable to address vision loss issues during the time of the COVID-19 pandemic and in the times to come. The COVID-19 outbreak has stressed the importance of reinforcing scientific cooperation. Multidisciplinary expertise should be involved in planning safe and efficient reorganization. Novel screening and diagnostic tests are emerging and they should be carefully considered as important strategic tools, not only within the ophthalmology department. Our practice will need to continuously integrate learnings in light of the SARS-CoV-2 renewed infection trends. Sharing experiences and strategies will be crucial while taking evidence-based decisions and guaranteeing the safety of patients and healthcare professionals.

Obviously, a universal approach would be neither feasible nor rational, since specific characteristics exist, at a regional and local level across Europe, in terms of COVID-19 epidemiology and national provisions, the availability of resources such as personal protective equipment (PPE) and facility’s staff, screening strategies, cataract pathways (day surgery vs. overnight staying), and healthcare payment systems (public coverage, reimbursement, and private insurance) [87].

To conclude, this report was prepared based on the current situation, but it is expected to evolve based on the ongoing pandemic and guidelines from national and international authorities. The “new normal” is raising many questions but now more than ever a vibrant exchange of strategies and experience is crucial to creating adaptive pathways that can be tailored to different institutions or clinics. None of the above-described procedures can guarantee a SARS-CoV-2 free facility but the aim is to create a perioperative pathway that is as safe as possible for patients and healthcare staff.
